# Recent insights into *Shigella:* a major contributor to the global diarrhoeal disease burden

**DOI:** 10.1097/QCO.0000000000000475

**Published:** 2018-09-06

**Authors:** Stephen Baker, Hao Chung The

**Affiliations:** a The Hospital for Tropical Diseases, Wellcome Trust Major Overseas Programme, Oxford University Clinical Research Unit, Ho Chi Minh City, Vietnam; b Centre for Tropical Medicine, Nuffield Department of Clinical Medicine, Oxford University, Oxford; c The Department of Medicine, University of Cambridge, Cambridge, United Kingdom

**Keywords:** diarrhoeal disease, epidemiology, genomics pathogenesis, *Shigella*

## Abstract

**Purpose of review:**

Diarrhoea is a major global health problem, and recent studies have confirmed *Shigella* as a major contributor to this burden. Here, we review recent advances in *Shigella* research; focusing on their epidemiology, pathogenesis, antimicrobial resistance, and the role of the gut microbiome during infection.

**Recent findings:**

Enhanced epidemiological data, combined with new generation diagnostics, has highlighted a greater burden of *Shigella* disease than was previously estimated, which is not restricted to vulnerable populations in low-middle income countries. As we gain an ever more detailed insight into the orchestrated mechanisms that *Shigella* exploit to trigger infection, we can also begin to appreciate the complex role of the gut microbiome in preventing and inducing such infections. The use of genomics, in combination with epidemiological data and laboratory investigations, has unravelled the evolution and spread of various species. Such measures have identified resistance to antimicrobials as a key contributor to the success of specific clones.

**Summary:**

We need to apply novel findings towards sustainable approaches for treating and preventing *Shigella* infections. Vaccines and alternative treatments are under development and may offer an opportunity to reduce the burden of *Shigella* disease and restrict the mobility of antimicrobial resistant clones.

## INTRODUCTION

Diarrhoea is a major global health issue. It accounts for approximately 1.3 million deaths each year, of which 500,000 are young children worldwide [1,2]. Despite the impressive reduction in diarrhoea-associated mortality over the past decade, there are still ∼950 million diarrhoea cases occurring in children less than 5 years annually [1]. This burden is mainly felt by low and middle-income countries in Asia and Africa, where accessibility to clean water, good nutrition, sustained sanitation, and healthcare is restricted. Tackling diarrhoea is complicated as the disease is caused by an array of bacterial, viral, and parasitic pathogens. Although improved sanitation has a major impact on lowering the incidence of all aetiologies, other public health measures, including appropriate treatment, education, and immunization remain crucial in furthering this success. Vaccines against rotavirus, the most common childhood diarrhoeal pathogen, are effectively alleviating diarrhoeal burden [3]. However, this global reduction in rotavirus disease is raising the profile, as well as the proportional burden, of other pathogens. This is particularly pronounced for bacterial agents such as *Shigella*, for which there is no licensed vaccine and treatment options become dwindling due to increasing resistance to key antimicrobials [4].

Recent estimates attribute *Shigella* to cause ∼125 million diarrhoeal episodes annually [5], leading to around 160 000 deaths, with a third of these associated with young children [1]. *Shigella*, along with enterotoxigenic *Escherichia coli* (*E. coli*), were identified as the predominant bacterial diarrhoeal pathogens in paediatric populations of South Asia and sub-Saharan Africa [6,7]. This research, the Global Enteric Multicentre Study (GEMS), also revealed that *Shigella* was the most prevalent aetiology in children aged 2 to 5 years who experienced diarrhoea. Reanalysis of the GEMS samples using quantitative molecular diagnostics suggested that *Shigella*-induced burden may actually be twice as high as previously estimated, ranking it as the most common detected pathogen [8^▪▪^]. Therefore, *Shigella* are a major contributor to the global diarrhoea burden and are arguably, given the associated disease severity and increasing antimicrobial resistance, the principal bacterial cause of sustained endemic diarrhoea. In the scope of this review, we highlight recent insights into the biology of *Shigella* and the disease that it causes, focusing on its pathogenesis, interaction with the microbiome, and the epidemiology of shigellosis. 

**Box 1:**
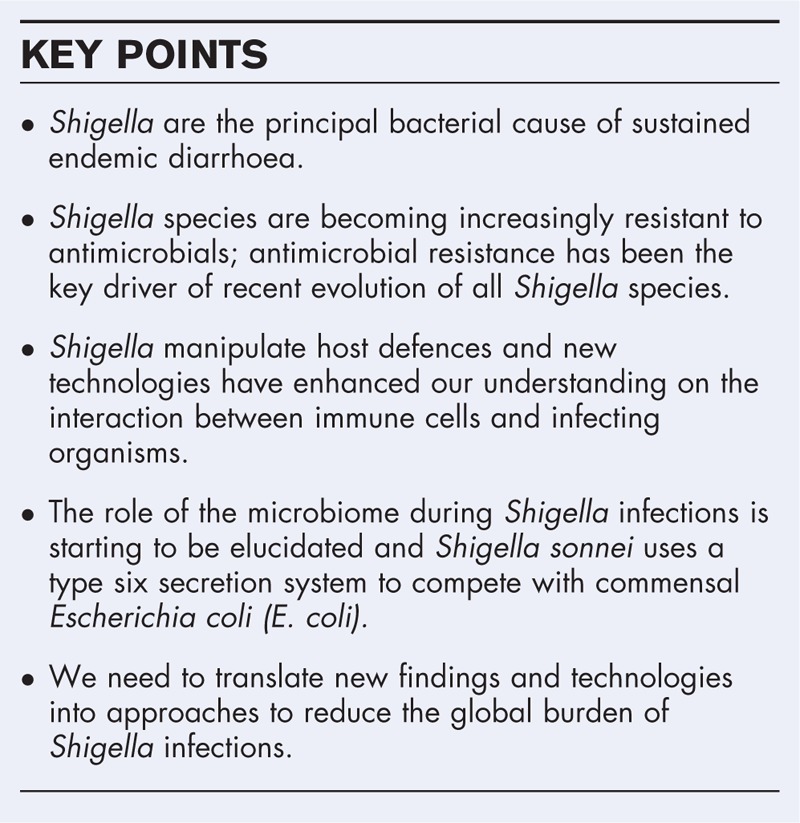
no caption available

## *SHIGELLA* PATHOGENESIS: THE BALANCE BETWEEN VIRULENCE AND PROTECTION

*Shigella* is a member of the Gram-negative Enterobacteriaceae family, and current classification divides the genus into four species based on serological typing: *S. dysenteriae*, *S. boydii*, *S. flexneri* and *S. sonnei*. Ingestion of *Shigella*, which typically has a low infectious dose, commonly results in an aggressive watery or mucoid/bloody diarrhoea. This clinical presentation is a direct consequence of *Shigella* invasion and destruction of the large intestinal epithelium. Briefly, the bacterium crosses the epithelium via M cells, and induces phagocytosis by macrophages in the submucosa. *Shigella* quickly activate macrophage death and interact with the epithelium's basolateral surface, triggering its uptake through the reorganization of host cell cytoskeleton [9–11]. Once inside the epithelial cell, they again lyse the surrounding phagosome and replicate, before disseminating intracellularly to adjacent cells using actin polymerization [12,13]. Central to this well-choreographed pathogenesis is the large virulence plasmid (more than 200 kbp), which encodes the syringe-like type three secretion system (T3SS) and an arsenal of effector proteins, including several invasion plasmid antigens (Ipas) [14–16]. Various reviews have been dedicated to detail *Shigella*'s pathogenesis [17,18] as well as to elucidate the role of each virulence factor [19^▪^].

Survival within host cells poses monumental challenges unmet by the free-living *E. coli* cousins, namely the detection and elimination by the host immune system. Decades of extensive research has portrayed *Shigella* as a master of survival, maintaining the subtle balance between virulence and immune protection. A classic example is the modulation of O-antigen (OAg) chain length in *S. flexneri*. Chromosomal *wzz* produces short-chain OAg to maximize the T3SS machinery's exposure to host cells whereas the pHS2 counterpart promotes long-chain OAg to mask *S. flexneri* from serum complement killing [20,21]. *S. sonnei* utilizes a different strategy to attain this same effect. It possesses a unique capsule made of OAg polysaccharides, decreasing its invasiveness in return for increased protection [22]. In addition, *Shigella* is particularly adept in subversion of the host immune response, targeting both the innate and adaptive systems [18,23]. Specifically, it is known to invade T lymphocytes via T3SS and arrest their migration in lymph nodes [24], and B lymphocytes are targeted for apoptosis via interaction with the T3SS effector IpaD [25]. These potentially deprive the human host to mount an effective and prolonged adaptive immune response. The initial process upon *Shigella* infection is the induction of macrophage pyroptosis, allowing the release of invading bacteria but compromising its survival by igniting a proinflammatory state [17]. Recently, IpaD was shown to mediate a noninflammatory macrophage apoptosis, thus trapping the pathogen within apoptotic bodies [26^▪^]. It is proposed that these parallel pathways are complementary to balance the trade-off between infectiousness and immune evasion. This same theme also underlies the functions of the IpaH family, a bacterial E3 ubiquitin ligase of research interest in recent years [27]. This enzyme catalyses the ligation of ubiquitin to target eukaryotic host proteins, usually designating them for degradation via proteasomes. *Shigella* carries numerous *IpaH* genes (situated both on chromosome and the virulence plasmid), which potentially affect ubiquitination in differing protein substrates [28]. Indeed, IpaH7.8 targets glomulin for proteolysis, thus indirectly activating inflammasomes and leading to macrophage pyroptosis [29]. In contrast, IpaH1.4 and IpaH2.5 were shown to suppress the NF-κB immune signalling by interfering with the linear ubiquitin chain assembly complex (LUBAC) machinery [30^▪▪^]. Immune suppression is also achieved through the IpaH9.8-mediated destruction of interferon-induced guanylate-binding proteins (GBPs), and this circumvents the host's cell-autonomous defence against intracellular microbes [31^▪▪^]. In addition, this same degradation is also essential in promoting cell-to-cell dissemination in *Shigella* infection [32^▪^]. The maintenance of the large virulence plasmid comes with a significant metabolic cost, which could be detrimental to *Shigella*'s survival in resource-limited environments outside the host. It may counteract this expenditure by integrating pINV into the chromosome, thus downregulating the expression of virulence genes. This phenomenon has been observed *in vitro* during *S. flexneri*'s growth at environmental temperatures, and reversible pINV excision restores its virulence at 37^o^C [33^▪^].

## NEW FRONTIERS: *SHIGELLA*'S INTERACTION WITH THE GUT MICROBIOME

Until recently, the focus of *Shigella* pathogenesis research has been on its interaction with the human host, and this overlooks the roles of the heterogenous colonic landscape and its coinhabiting microbial communities. Use of innovative 3D fluorescent imaging and analyses help track *S. flexneri* journey *in vivo*, revealing that the pathogen targets colonic crypts during the early phase of infection [34]. These crypts house the intestinal stem cells at their base and harbour their own crypt-specific core microbiota (CSCM) [35]. Though *Shigella*'s invasive zone rarely reaches the crypt base to disrupt stem cells progeniture, its interaction with the CSCM and indirect consequences on gut health remain unexplored. Successful invasion requires *Shigella* to overcome two gut-specific barriers: the microbiota and the mucus layer [36]. Colonic commensals could prevent pathogen proliferation by either direct competition for space and nutrient, secretion of antimicrobials, or modulation of immune response. Additionally, *S. sonnei*, but not *S. flexneri*, harbours an active type VI secretion system (T6SS), which kills co-inhabiting *E. coli* at infecting tissues [37^▪▪^]. A defective T6SS phenotype leads to reduced persistence in the colon, indicating that this apparatus is crucial for *S. sonnei* to overcome *E. coli*-established colonization resistance.

Two interesting questions remained insufficiently answered regarding *Shigella*'s relationship with the gut microbiome: Which microbial communities are protective of *Shigella* infection in humans? And how does the human gut microbiome respond to a *Shigella* infection? Breakthroughs in sequencing, commonly employed as 16S rRNA profiling and shotgun metagenomics, have allowed an interrogation of microbial communities at the molecular level. In order to investigate the first question, it is important to evaluate the subjects clinically and microbiologically, pre and postinfection. However, data of such resolution is realistic from human challenge and longitudinal cohort studies, which are scarce. Previous immunization trials in macaques showed that *Prevotella*-rich microbiota was associated with asymptomatic infections upon challenge with wildtype *S. dysenteriae*[38]. Nonetheless, this effect is only apparent in one macaque genotype, prompting the contribution of other host factors. *Prevotella* are considered biomarkers for plant-based diets rich in fibre [39], and low fibre uptake prompts the gut microbiota to digest host's mucus glycoprotein [40]. This may result in rapid degradation of the mucus barrier, ultimately leading to increased susceptibility to invasion by bacterial pathogens, such as *Shigella*. Besides, the abundance of *Prevotella* species was shown to be negatively correlated with the copy number of *Shigella*/EIEC specific *IpaH* in diarrhoeal stools [41]. These studies suggest that *Prevotella*-rich microbiota is potentially protective for *Shigella* infections, but this will require further investigations. Regarding the second question, an examination on the diarrhoeal microbiome in Vietnamese young children indicated that the gut microbiota's response to *Shigella* infections is varied and nonspecific to the pathogen [42^▪^]. Instead, factors such as age, nutritional status, breastfeeding practice, and type of infection (virus/bacteria) are more indicative of the initial gut microbiota structures upon diarrhoea.

## CHANGING EPIDEMIOLOGY AND THE CHALLENGE OF MULTIDRUG RESISTANCE

The four *Shigella* species and their various serotypes have differing geographical distribution and epidemiological significance. *S. boydii* infections are uncommon outside the Indian subcontinent, and there is currently limited epidemiological data regarding this species. *S. dysenteriae*, specifically *S. dysenteriae* 1, was the causative agent of multiple fatal dysentery epidemics since its first isolation in 1897 [43]. However, this species is rarely being isolated in current surveillance, and its decline is likely due to improvements in sanitation and antimicrobial access [5,44]. The current global epidemiological burden for shigellosis is attributed to two species, *S. flexneri* and *S. sonnei*, which were conventionally associated with developing and developed regions, respectively. Nevertheless, recent evidence points to the emergence of *S. sonnei* in economically transitional states, effectively replacing *S. flexneri* to become the predominant shigellosis aetiology [45]. This species replacement phenomenon is repeatedly documented in many countries in Asia, such as Vietnam [46], Thailand [47], and Bangladesh [48]. This shifting epidemiology is again reflected in the *Shigella* collection from GEMS, in which the authors argued that a quadrivalent vaccine targeting *S. sonnei*, *S. flexneri* 2a, *S. flexneri* 3a, and *S. flexneri* six is desired to provide sufficient coverage and protection against shigellosis in endemic regions [49].

Studies combining epidemiological and high-resolution pathogen's genomic data are increasingly common. This approach has untangled the evolutionary history and ecological dynamic of various *Shigella* species. Specifically, phylogenomic analyses of more than 300 temporally and spatially diverse *S. dysenteriae* one sequences proposed its existence as early as since the 18th century [50^▪▪^]. Intercontinental transmissions heightened quickly since the late 19th and throughout the 20th century, and recent waves of introductions from South Asia to Africa were responsible for multiple epidemics. Similarly, existing *S. sonnei* have been shown to likely descend from a common ancestor in the 17th century in Europe, and the expansions of the two main lineages (II and III) have led to their global dissemination since the 20th century [51,52^▪^]. These studies emphasize a pattern recognized between many *Shigella* species, whereby organisms are mobilized globally and then form localized endemic transmission. This is exemplified at the genomic scale by *S. sonnei*'s introduction and subsequent establishment in Vietnam [53] and Latin America [52^▪^]. Alternatively, due to its low infectious dose and human-restricted nature, *Shigella* is able to induce sustained transmissions in close contact communities. *Shigella* causing several outbreaks in Orthodox Jewish communities in the United Kingdom, mainland Europe and North America are genetically closely related and clustered with those sampled in Israel, forming a single population diverging since the late 1980s [54^▪^]. In the United Kingdom, domestic *Shigella* transmissions have been exclusively noted in MSM communities, resulting in at least four discrete *S. sonnei* transmission chains with low genetic diversity [55].

Shigellosis is a self-limiting disease, with patients usually fully recovered within 7–10 days. However, the infection is known to cause potential complications, most severely encephalopathy [56]. Therefore, antimicrobial treatment is recommended to prevent further complications, reduce diarrhoeal output, and limit postsymptomatic faecal shedding [57,58]. However, the appropriate choice of antimicrobials is subject to debate, and no agent emerges to be superior clinically [59^▪^]. Unfortunately, resistance to antimicrobials appears to arise comparatively effortlessly in *Shigella* and may be a consequence of an unrestricted barrier for horizontal gene transfer between *Shigella* and other Enterobacteriaceae. Sulphonamide, tetracycline, streptomycin, and chloramphenicol were initially deployed to treat *Shigella* infections, but organisms that were nonsusceptible to all four antimicrobials emerged during the late 1950s. This phenotype was later determined to be conferred by small plasmids, such as spA in *S. sonnei*. Ampicillin, and later co-trimoxazole were used as alternatives, but these soon again met resistance in the 1980s [60]. Resistance to these agents could have facilitated the expansion and global spread of fit clones, exemplified by the integration of Tn7 transposon (encoding *dfrA1* for trimethoprim resistance) in successful *S. sonnei* lineage III and *S. dysenteriae* one lineage IV [50^▪▪^,^51^]. Subsequent use of a quinolone, nalidixic acid, led to rapid and independent developments of resistance in endemic areas by 2000. The current recommended first-line treatment for shigellosis is fluoroquinolones, such as ciprofloxacin, and these quickly become the mainstay prescription for shigellosis as well as acute diarrhoea in endemic regions [58]. Mainly due to its common use, resistance to ciprofloxacin is widespread among *Shigella* retrieved globally since the turn of this century, and Asia serves as a likely reservoir for the rise and spread of resistant organisms [61]. Specifically, ciprofloxacin-resistant *S. sonnei* has evolved as a single clone, most likely in South Asia, before spreading internationally to Southeast Asia and Europe [62^▪▪^]. Such resistance relies on gradual accumulation of the triple mutations in chromosomal *gyrA* and *parC*. Additionally, horizontally transferred elements could help shape and establish emerging resistant clones. Recent years have witnessed a stark increase in azithromycin resistant *S. flexneri* 3a in MSM communities worldwide, which is caused by the propagation of a single sublineage of this species since 1998 [63]. Resistance to azithromycin is induced by the mobile plasmid pKSR100, which was recently shown to be acquired in separate *S. sonnei* and *S. flexneri* 2a populations [64^▪▪^]. This greatly facilitated new transmission chains, creating multiple co-circulating resistant *Shigella* epidemics in the United Kingdom's MSM community.

The evolutionary pressure created by antimicrobial usage fuels new resistances among *Shigella*, and globalization has enhanced an unprecedented mobility of this human restricted pathogen. In the present and coming age when multidrug resistance (MDR) is becoming the norm, much remains unanswered on how *Shigella*'s state-of-resistance translates to clinical care. A recent study on paediatric diarrhoea in Vietnam found that hospitalization length for *Shigella* infected patients is similar regardless of the ciprofloxacin susceptibility profile of the associated organism [65^▪^]. Therefore, MDR likely poses a more significant threat to certain high-risk cohorts, including the malnourished, the elderly, and the immunocompromised. The latter is of increasing concern for MDR *Shigella* is surging in HIV-positive MSM, who present more severe clinical symptoms and require effective antimicrobial therapy [66].

## CONCLUSION

The combination of larger epidemiological studies, more sophisticated in-vitro technologies, and genomics have provided unprecedented insights into the success of the genus *Shigella*. These could be invaluable to the development of future vaccines and alternative therapies. Namely, *Shigella* vaccines should account for the pathogen's numerous tricks to manipulate the immune response as well as the rapidly changing epidemiology. Novel therapies could benefit from the detailed portrayal of *Shigella*'s pathogenesis and interactions with the gut microbiota. These tools need to be accelerated to stem the tide of increasingly antimicrobial resistant *Shigella* clones. *Shigella* research has reached a pivotal state, and we now need to apply our knowledge, technologies and experience to reduce the disease burden of this bacterial pathogen.

## Acknowledgements

None.

### Financial support and sponsorship

S.B. is a Sir Henry Dale Fellow, jointly funded by the Wellcome Trust and the Royal Society (100087/Z/12/Z).

### Conflicts of interest

There are no conflicts of interest.

## REFERENCES AND RECOMMENDED READING

Papers of particular interest, published within the annual period of review, have been highlighted as:

▪ of special interest
▪▪ of outstanding interest
